# Small steps or giant leaps for male-killers? Phylogenetic constraints to male-killer host shifts

**DOI:** 10.1186/1471-2148-7-238

**Published:** 2007-11-29

**Authors:** Matthew C Tinsley, Michael EN Majerus

**Affiliations:** 1 School of Biological and Environmental Sciences, University of Stirling, Stirling, FK9 4LA, UK; 2 Department of Genetics, University of Cambridge, Downing Street, Cambridge, CB2 3EH, UK

## Abstract

**Background:**

Arthropods are infected by a wide diversity of maternally transmitted microbes. Some of these manipulate host reproduction to facilitate population invasion and persistence. Such parasites transmit vertically on an ecological timescale, but rare horizontal transmission events have permitted colonisation of new species. Here we report the first systematic investigation into the influence of the phylogenetic distance between arthropod species on the potential for reproductive parasite interspecific transfer.

**Results:**

We employed a well characterised reproductive parasite, a coccinellid beetle male-killer, and artificially injected the bacterium into a series of novel species. Genetic distances between native and novel hosts were ascertained by sequencing sections of the 16S and 12S mitochondrial rDNA genes. The bacterium colonised host tissues and transmitted vertically in all cases tested. However, whilst transmission efficiency was perfect within the native genus, this was reduced following some transfers of greater phylogenetic distance. The bacterium's ability to distort offspring sex ratios in novel hosts was negatively correlated with the genetic distance of transfers. Male-killing occurred with full penetrance following within-genus transfers; but whilst sex ratio distortion generally occurred, it was incomplete in more distantly related species.

**Conclusion:**

This study indicates that the natural interspecific transmission of reproductive parasites might be constrained by their ability to tolerate the physiology or genetics of novel hosts. Our data suggest that horizontal transfers are more likely between closely related species. Successful bacterial transfer across large phylogenetic distances may require rapid adaptive evolution in the new species. This finding has applied relevance regarding selection of suitable bacteria to manipulate insect pest and vector populations by symbiont gene-drive systems.

## Background

Reproductive parasites represent a diverse assemblage of maternally inherited microorganisms that induce aberrations in their hosts' reproductive biology in order to proliferate within arthropod populations. Their vertical transmission route greatly limits opportunities for interspecific transfer. However, phylogenies of hosts and parasites are generally not congruent, indicating that interspecific horizontal transfer events characterise the evolutionary history of these selfish genetic elements [1].

Two different modes of reproductive parasitism exist that aid the maintenance of these microbes in host populations. Some maternally inherited parasites bias host progeny sex ratios in favour of female offspring, through which they then achieve transmission (by feminization, parthenogenesis induction or male-killing); others modify sperm of infected males, in order to reduce the fitness of the uninfected females with which they mate (cytoplasmic incompatibility) [2]. Whilst *Wolbachia *is the best known, a variety of other microbial taxa has also evolved to manipulate arthropod reproduction, including microsporidia, gamma proteobacteria and members of the genera *Cardinium*, *Rickettsia *and *Spiroplasma *[2-5].

Individual symbioses between reproductive parasites and host species are generally short-lived relative to the speciation rate of hosts [6,7] (although exceptions exist [8]). Transience may be due to host resistance evolution [9] or alternatively some sex ratio distorters risk driving host extinction due to male shortage [10]. Long term persistence of parasite lineages therefore demands horizontal transfer to exploit new host species. It seems logical that horizontal transfer should be more common within species than between them. Nevertheless strong linkage disequilibrium usually exists between bacterial strains and host mitochondrial DNA, indicating that intraspecific horizontal transfer is exceptionally rare [11-14]; opportunities for transmission between species must thus be highly restricted. One exception is the close ecological contact between parasitoids and their hosts, which may facilitate transfer between wasps following oviposition in a common host [15,16] and between host and parasitoid [17,18].

Three potential routes exist for a species to acquire a reproductive parasite infection: direct transfer of infective material, introgressive hybridisation or co-speciation of host and bacterium. Phylogenetic studies indicate that the majority of symbioses originate from infective transfer: bacterial strains are usually considerably more closely related to each other than are their hosts [6,19,20]. However, such studies cannot generally distinguish whether transfer occurred directly between two hosts, or if unstudied intermediate species were involved (but exceptions are known [18]). Although rare, cases exist where high recent host speciation rates have enabled host-parasite co-speciation [8]. Furthermore, hybridisation between closely related hosts has permitted introgression of *Wolbachia *in at least two cases and may be responsible for others [12,21]. Whatever the transmission route, phylogenetic studies reveal that closely related reproductive parasite strains may specialise on closely related arthropod species; either reflecting symbiont adaptation to similar hosts or the frequency of horizontal transmission opportunities [6,22]. Once a reproductive parasite has infected a novel host, the probability of invasion will be determined by its ability to induce a reproductive phenotype, its vertical transmission efficiency and its virulence in the new species.

An increasing number of studies report experimental interspecific transfers of *Wolbachia *and other symbionts [23-28]; most of these transfers have been intra-generic. Transfection of the *Drosophila simulans *cytoplasmic incompatibility (CI) strain *w*Ri into *D. melanogaster *and parthenogenesis inducing (PI) *Wolbachia *within the *Trichogramma *genus both resulted in maternal transmission and expression of the original phenotype [23,25]. Host-shifts of increasing phylogenetic distance have had variable success. Whilst the *D. simulans w*Ri strain transmitted and induced CI in *Aedes albopictus*, a PI *Wolbachia *from *Muscidifurax uniraptor *transmitted but induced no phenotype in *D. simulans *[24,29]. Experimental transfer of *Drosophila *SROs (male-killers) generally led to establishment in other *Drosophila *species; however, in some cases male-killing ability was reduced and maternal transmission unstable [27].

The experimental transfer of reproductive parasites between host species is of practical as well as evolutionary interest. Due to their ability to spread deterministically through host populations, microbes such as *Wolbachia *have been proposed as potential gene drive systems to control insect pest population sizes and manipulate vector competence [30,31]. In addition to genetically engineering the symbionts concerned, such techniques may require infection of target species with well characterised bacterial strains from other hosts. Some successful transfers to vector species have recently been reported [32] but others have had less promising results [26].

There is phylogenetic evidence for horizontal transfer of bacteria between both closely and distantly related host species and an increasing number of reports demonstrating experimental transfection. However, only a minority of studies have attempted transfers outside the native genus and systematic research to investigate the effect of phylogenetic distance on the success of experimental infections is lacking. It also seems likely that there is a publication bias towards reports of successful transfection studies.

This study tested the hypothesis that increasing phylogenetic distance between native and novel hosts decreases the likelihood of reproductive parasites colonising a new species. The *Spiroplasma *male-killer of the coccinellid beetle *Adalia bipunctata *was employed because it has previously been demonstrated to transfer repeatably by injection [33]. The bacterium was injected into pupae of seven novel coccinellid species. Within the *Adalia *genus it was injected into both the native host *A. bipunctata *and its sister species *A. decempunctata*. We transfected five species outside the *Adalia *genus but within the Coccinellinae subfamily: *Coccinella septempunctata*, *Harmonia quadripunctata*, *Anisosticta novemdecimpunctata*, *Calvia quatuordecimguttata *and *Propylea quatuordecimpunctata*. We also injected *Exochomus quadripustulatus*, which lies in the Chilocorinae subfamily [34]. Finally, we injected the non-male-killing *Spiroplasma *symbiont of the aphid *Acyrthosiphon pisum *[35] into *A. bipunctata*. In each case three questions were investigated: could the bacterium establish an infection, could it transmit trans-ovarially and did it kill males in the novel host?

## Results

### Phylogenetic relationships between host species

**Figure 1:**
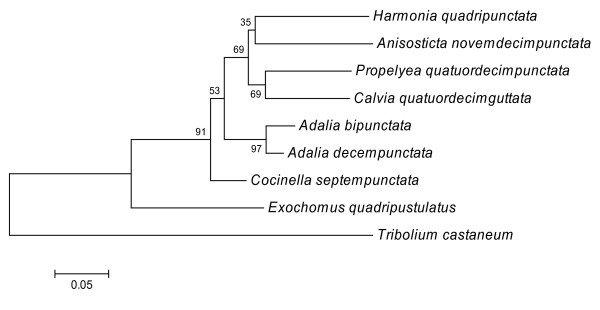
**Phylogenetic relationships of the coccinellid beetle species, derived from a combined 16S and 12S mitochondrial rDNA dataset**. Tree estimation procedures are described in methods. The results of 1000 bootstrap resampling replicates are given next to branches, branch lengths are proportional to genetic distance (base substitutions per site, see scale bar). *Tribolium castaneum *is included as an outgroup.

We produced 661 bp of 16S and 396 bp of 12S mitochondrial rDNA sequence; of the 1057 sites in our dataset, 386 were variable and 200 parsimony informative. The neighbour joining tree produced (Figure 1) broadly confirmed existing phylogenetic positions based on morphological characters [34].

### Parental Lines

The female parents of all species from which recipient pupae for injection were derived produced offspring sex ratios not significantly different from equity (Fisher exact tests for all 19 parents, P > 0.18). Negative PCR results demonstrated that no parents were infected by the bacterial taxa *Spiroplasma*, *Rickettsia*, *Wolbachia *or Flavobacteria.

### Adalia genus transfers

**Figure 2:**
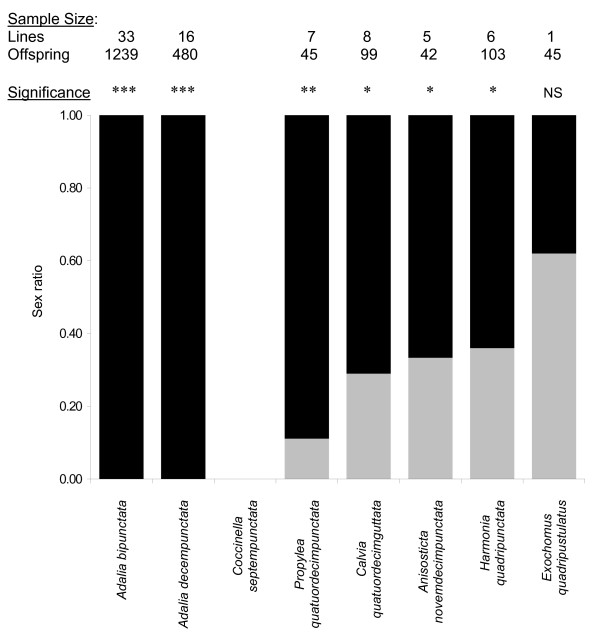
**Offspring sex ratios produced by females of each species injected with the *A. bipunctata Spiroplasma *male-killer**. Average proportions of female and male offspring are represented by black and grey shading respectively. Species bars are shown in order of increasing genetic distance from *A. bipunctata *(left to right). Number of matrilines and total number of offspring per species are given above each bar. The results of Fisher exact tests for the significance of sex ratio deviations away from equity are indicated above bars by asterisks. *Coccinella septempunctata *females produced no offspring.

**Figure 3:**
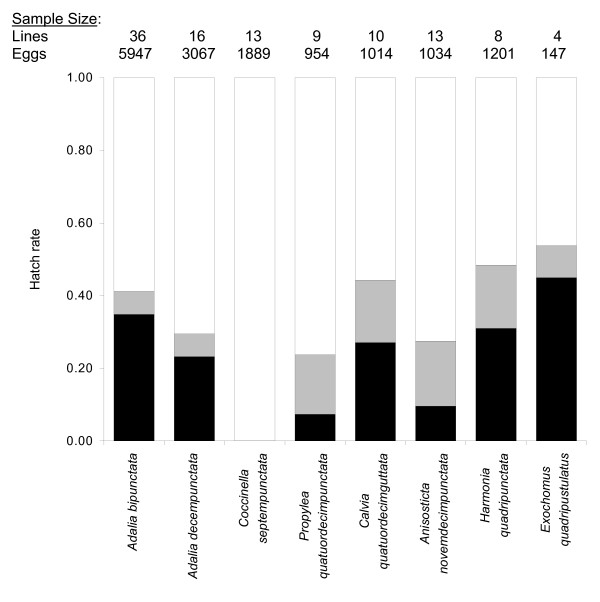
**Hatch rates of eggs laid by females of the species injected with the *A. bipunctata Spiroplasma *male-killer**. Average proportions of eggs that hatched are shown by black bars. Proportions of unhatched eggs are divided between those that showed no signs of embryonic development (in white) and those that became grey and died shortly before hatching (in grey). Species bars are shown in order of increasing genetic distance from *A. bipunctata *(left to right). Number of matrilines and total number of offspring per species are given above each bar.

In total 33 *A. bipunctata *control females that had been injected with their native *Spiroplasma *were bred. They produced no male offspring (Figure 2). Mean progeny per line was 37.5 (range 4 – 95); in all, 1239 females resulted. Matriline sex ratios differed significantly from equity in 28 cases (Fisher exact test, Bonferroni corrected for 33 multiple comparisons). All females laying substantial numbers of eggs had hatch-rates below 50% (mean = 0.32) indicating that male-killing was taking place (Figure 3). An F_1 _female from each line was confirmed infected with *Spiroplasma *by PCR. The microinjection technique was thus both effective and replicable; infectivity of all homogenate samples used to inject novel species was confirmed.

Complete male-killer expression also occurred in *A. decempunctata *transfected lines. Sixteen females were bred, which all solely produced female offspring, 480 in total (Figure 2). Mean offspring number per line was 30.0 (range 4 – 48), 14 progeny sex ratios differed significantly from equity (Fisher exact test, Bonferroni corrected for 16 multiple comparisons). Low hatch rates were again recorded (mean = 0.27) and one offspring from each female was demonstrated *Spiroplasma *infected by PCR.

### Inter-genus transfers

**Figure 4:**
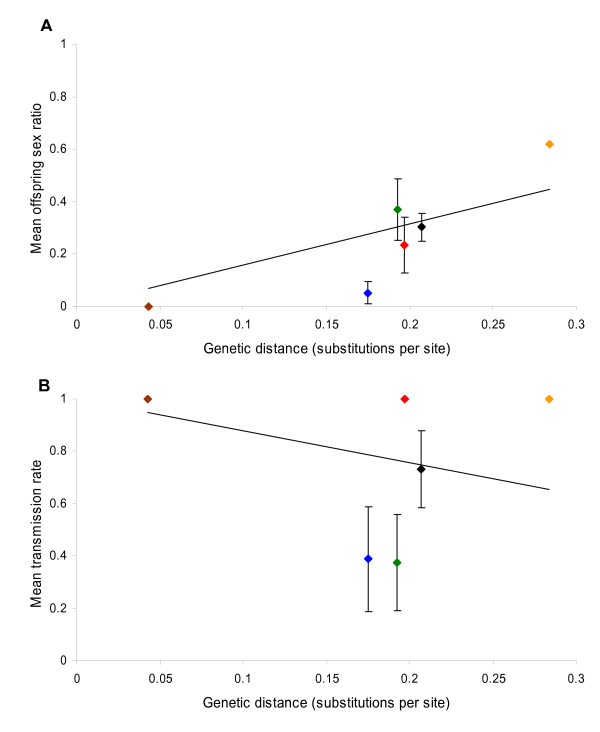
**Relationship between host shift genetic distance and bacterial traits in novel species**. Figure 4a – the relationship between interspecific transfer distance and the degree of host offspring sex-ratio distortion achieved by the male-killer (proportion male); the correlation is significant (see text). Figure 4b – the relationship between host shift genetic distance and vertical transmission to host offspring (proportion infected); the correlation is not significant (see text). Data points represent means for each species. A least squares best fit line fitted through the origin (4a) and through 1.0 (4b) is shown for each graph. All interspecific transfections are represented apart from *C. septempunctata *(for with no offspring resulted): brown, *A. decempunctata*; blue, *P. quatuordecimpunctata*; green, *C. quatuordecimguttata*; red, *A. novemdecimpunctata*, black; *H. quadripunctata*, orange; *E. quadripustulatus*. Error bars show standard errors, where no bar is visible variance was zero (apart from *E. quadripustulatus *for which n = 1).

PCR tests at least six weeks after injection detected *Spiroplasma *infections in 100% of the parental females for all six species outside the *Adalia *genus (total n = 70; n per species 8 – 15). The bacterium therefore survived and replicated in these novel hosts. However, inter-genus transfers generally resulted in incomplete sex ratio distortion and impaired vertical transmission. These factors could not be assessed in *C. septempunctata *for which all females (n = 13) injected with the *Spiroplasma *were sterile (see below). In total, across the five remaining species 27 fertile females produced 334 offspring, 66% were female. All species exhibited significantly female-biased sex ratios except for *E. quadripustulatus *(Figure 2). Many females of the five species displayed low egg hatch rates, fecundity and survivorship. However, except in *C. septempunctata*, a considerable proportion of eggs hatched or reached late embryonic development (became grey); thus low hatch rates did not result from infertility and were indicative of male-killing (Figure 3). Pooling across species, sex ratio distortion was significantly impaired in females outside the native genus (mean sex ratio = 0.26 ± 0.05 SEM, n = 27) in comparison to transfers to congeneric *A. decempunctata *hosts (mean sex ratio = 0.0 ± 0.0 SEM, n = 16) (Wilcoxon W_(n = 27,16) _= 208; P < 0.001). Furthermore, a significant positive correlation between mean offspring sex ratio and genetic distance of transfection existed across species, indicating that progressively more distant host shifts reduced the degree of sex ratio distortion the bacterium achieved (Figure 4a) (r_s(n = 6) _= 0.829; P < 0.042).

*Spiroplasma *transmission rates in novel host-parasite associations were assessed by PCR (Table 1). Vertical transmission was perfect in *A. bipunctata *and *A. decempunctata*. However, only in two of the five fertile species outside the *Adalia *genus (*A. novemdecimpunctata *and *E. quadripustulatus*) did all offspring inherit the bacterium. *Calvia quatuordecimguttata *females behaved inconsistently: three females transmitted the *Spiroplasma *to all offspring tested (16 females, 1 male), whereas five females transmitted it to none (20 females, 14 males). *Harmonia quadripunctata *females were similarly variable: one female produced a significant sex ratio bias of 0.17 (n = 18) and transmitted to all offspring tested (7 females, 1 male), whereas another whose sex ratio was 0.47 (n = 53) transmitted to only 19% of progeny (19 females, 23 males tested). *Propylea quatuordecimpunctata *females produced a strongly female-biased sex ratio (0.11, n = 45) but surprisingly only 15% of offspring were infected when assayed as adults (n = 20). The bacterial transmission rate in hosts following inter-generic transfers (mean = 0.61 ± 0.09 SEM, n = 26) was significantly lower than that in congeneric *A. decempunctata *females (mean = 0.0 ± 0.0 SEM, n = 16) (Wilcoxon W_(n = 26,16) _= 483 P < 0.001). However, considering all interspecfic transfections, the negative relationship between mean transmission rate and genetic distance was not significant (Figure 4b) (r_s(n = 6) _= -0.213; P = 0.686). In three non-*Adalia *species (*H. quadripunctata*, *A. novemdecimpunctata *and *E. quadripustulatus*) a considerable percentage of male progeny were infected (Table 1). Pooling across all inter-genus transfers 46% (n = 72) of male offspring survived embryonic infection and carried the *Spiroplasma *as adults.

**Table 1:** Transmission efficiency of the *A. bipunctata Spiroplasma *male-killer following injection into different coccinellid species.

Species	N	Females		Males	
		Infected	Uninfected	Infected	Uninfected

*A. bipunctata*	33	33	0	-	-
*A. decempunctata*	16	16	0	-	-
*C. septempunctata*	-	-	-	-	-
*H. quadripunctata*	6	20	18	11	22
*A. novemdecimpunctata*	5	12	0	12	0
*C. quatuordecimguttata*	8	16	20	1	14
*P. quatuordecimpunctata*	6	3	14	0	3
*E. quadripustulatus*	1	10	0	9	0

Offspring of injected females were tested by PCR for *Spiroplasma *infection when adults. Numbers of infected males and females from each species are shown separately. The *Adalia *species produced no males. *Coccinella septempunctata *produced no fertile eggs, but clutches did carry the bacterium. The number of matrilines from which the offspring tested were derived is shown (N).

*Coccinella septempunctata *responded unusually to *Spiroplasma *injection: all females were sterile. Thirteen injected females laid eggs, however not one hatched (n = 1889, mean eggs per female 145.3). All eggs remained yellow and showed no signs of embryonic development. Each female was mated to at least two independent males to ensure male infertility was not the cause. Egg clutches laid by these females carried the *Spiroplasma *(n = 10). Subsequently, 14 further females were produced that had been injected with homogenate of uninfected *A. bipunctata *females. Ten were fertile (71%) with hatch rates ranging from 0.07 to 0.81. The mean hatch rates of females injected with uninfected and infected homogenate (0.29 ± 0.07 SEM and 0.00 ± 0.00 SEM respectively) differed significantly (t-test, arcsin transformed data, t_(*v *= 25)_= 4.41; P = 0.002).

In addition, a non-male-killing *Spiroplasma *from the aphid *Acyrthosiphon pisum *was injected into *A. bipunctata *pupae. One egg clutch was tested for *Spiroplasma *from each of 50 injected females; none was infected. These 50 females were all similarly uninfected when they were killed and tested by PCR at least 5 weeks post-injection. Four individuals tested within two days of injection shortly after pupal emergence were all positive.

## Discussion

This study investigated the host specificity of a reproductive parasite, the male-killing *Spiroplasma *of the coccinellid beetle *Adalia bipunctata*. We injected it into seven novel species and investigated bacterial transmission and sex ratio distortion. We tested the hypothesis that the phylogenetic distance between native and potential novel species has constrained host-shifts during male-killer evolution. The full male-killing phenotype was retained following transfers within the native genus, but in more distantly related species inefficient transmission or incomplete sex ratio distortion occurred.

Across every species tested injections always established an infection; bacteria were thus consistently able to survive the immune response associated with host injury and interact with cells in novel species. *Wolbachia and Spiroplasma *reproductive parasites do not naturally stimulate their hosts' immune systems, but *Spiroplasma *densities do fall following artificial immune challenge [36,37]. This male-killer was therefore probably able to maintain immune evasion in novel hosts. Immune evasion may be aided if bacteria reside permanently or at times within host cells, where the antimicrobial response does not operate. The failure of the inter-order transfer of the non-male-killing *A. pisum *symbiont suggests that *Spiroplasma *tolerance to novel host environments is not without limit.

Bacteria were detected in eggs and offspring of all novel species following interspecific transfer between coccinellids. Thus no qualitative constraint to trans-ovarial transmission exists at this phylogenetic level. Bacterial transmission in novel species suggests absence of very tight specificity in the molecular interactions required, or considerable evolutionary conservation of the host molecules exploited. However, the efficiency and consistency of maternal inheritance did vary between species. Whilst transmission was perfect in *A. bipunctata *and *A. decempunctata*, in some cases outside the native genus it was reduced. Overall there was no consistent correlation between host-shift genetic distance and transmission rate; indeed no uninfected offspring were detected in two of the more distantly related species *A. novemdecimpunctata *and *E. quadripustulatus*.

The *Spiroplasma *maintained an ability to distort sex ratios following all inter-specific shifts across the native sub-family (Coccinellinae). However, as genetic distance of the novel host increased the degree of sex ratio distortion achieved by the bacterium fell. Whilst male-killing did occur outside the *Adalia *genus, in most other species infected males were produced. Therefore, in some cases, whilst the bacterium did transmit, male-killing failed. To distort sex ratios male-killers must interact with host sex determination mechanisms or male-specific gene products [38] and also be present in high enough density to kill male embryos [39,40]. It is possible that bacterial density in distantly related species was lower than in the native host, as has been reported following other interspecific transfections [23,41]. If correct, this hypothesis could explain the variable and reduced transmission rates as well as inefficient male-killing observed in some novel hosts. Phylogenetic variation in the molecules used to detect or kill males (such as sex determination pathways) may provide a further explanation for low male-killing penetrance in novel species. Male-killers require the ecological advantage provided by male embryo death to spread in host populations [42], therefore incomplete sex ratio distortion (due to low transmission or inefficient male-killing) would inhibit invasion of novel host species.

We did not set out to investigate the virulence of this bacterium in novel species. However, we found all *C. septempunctata *hosts were sterile following injection of the *Spiroplasma*. It is possible this resulted from specific pathogeneic effects of infection, or an inability of the bacterium to selectively kill only male embryos in this species. Strong selection acts on vertically transmitted parasites to reduce virulence costs on their hosts [43]. Future studies might assess pathogenic effects of reproductive parasites following host shifts (eg [41]). Increased virulence would represent another factor reducing the probability of population invasion following interspecific transfers.

Our study has assessed the average performance of a reproductive parasite in a panel of novel host species. There was variation in male-killing and transmission between individuals in most transfected species. It therefore remains possible that infections that kill males and transmit efficiently may occasionally establish following transfers beyond the genus level. Such events, however rare, might provide opportunities for parasites to successfully invade. In addition, we have not assessed transmission and sex ratio distortion past the F_1 _generation. Nevertheless, our data provide controlled comparisons of the likelihood of reproductive parasites retaining high fitness and establishing in a population following host shifts. A previous study transferred *A. novemdecimpunctata*'s native male-killing *Spiroplasma *into *A. bipunctata *and also reported incomplete male-killing [20]. Therefore our data might have general relevance beyond this particular male-killing parasite. The manipulations of reproductive parasites employing other phenotypes may well be adversely affected by host shifts in a similar phylogenetically dependent manner.

We have demonstrated clear phylogenetic constraints to the interspecific movement of a male-killing bacterium. Molecular studies suggest that natural reproductive parasite host-shifts have occurred more frequently between closely related hosts [6,22]. The present data indicate this may be the result of similarity in host physiology or genetics. Given molecular evidence that closely related bacterial strains do infect distantly related species [19,20], we propose two scenarios for horizontal interspecific movement. Firstly, male-killers may make phylogenetically 'small steps' with relative ease whilst retaining efficiency of the original reproductive manipulation. Secondly, if phylogenetic 'giant leaps' occur, then persistence in novel hosts may require rapid bacterial evolution to improve sex ratio distortion, transmission or virulence.

One widely discussed application for bacterial reproductive parasites is in the control of insect pest or disease vector populations [30,44,45]. Releases of CI *Wolbachia *strains might be used to reduce pest species population sizes [31,46]. Alternatively, the invasive nature of CI *Wolbachia *might be exploited to drive trans-genes conferring refractoriness to human disease transmission through insect vector populations [30]. The technical and practical aspects of these techniques have yet to be fully resolved. However, most require the introduction of well characterised symbionts into the target species. Such experimental transfers have indeed been achieved (eg [32]). Nevertheless our study emphasises that except in the case of small phylogenetic steps, ensuring high bacterial transmission fidelity and phenotype retention in field conditions may be a considerable challenge. Engineering native infections of the species concerned may be more generally successful than introducing novel bacteria.

## Conclusion

This study indicates that constraints of host physiology or genetics have limited opportunities for successful horizontal transfers during reproductive parasite evolution. We demonstrated that the *Adalia bipunctata *male-killing *Spiroplasma *was only able to maintain full transmission and offspring sex ratio distortion after being injected into hosts within the native genus; more distant transfers resulted in low-fitness infections. Reproductive parasites thus exhibit a considerable degree of evolutionary specialisation on their natural host. Host-shift genetic distance was significantly correlated with sex ratio distortion ability but its relationship with transmission rate was inconsistent and not significant. This finding suggests the possibility that the reproductive manipulations of these parasites may be more generally sensitive to host shifts than their transovarial transmission. Our data offer experimental support for phylogenetic studies which indicate that host-shifts are relatively frequent between closely related species.

## Methods

### Ladybird Material

Donor individuals were *A. bipunctata *females carrying this species' native male-killing *Spiroplasma*, which displays perfect vertical transmission in the laboratory and has previously been shown infective by injection [33]. Donors were F_1 _progeny of parents collected in Stockholm. Novel recipient species were all collected close to Cambridge, UK. Offspring of field collected females were reared in the laboratory to pupal stage using standard techniques [47]. Parental females of each species from which recipient pupae were derived were assayed by PCR for the presence of any known coccinellid sex ratio distorting bacteria (see below) and offspring sex ratios were recorded to ensure that they were equitable in the generation prior to injection. *Adalia bipunctata *is host to at least three other male-killers, a *Rickettsia *and two strains of *Wolbachia *[48,49], furthermore *A. decempunctata *carries a male-killing *Rickettsia *[50], *A. novemdecimpunctata *carries a male-killing *Spiroplasma *[20] and *H. quadripunctata *has a male-killing flavobacterium (Majerus unpublished). None of these symbionts was present in any recipient pupae.

### Spiroplasma transfection

Injection techniques followed Tinsley and Majerus [20]. Briefly, *A. bipunctata *abdomen homogenate was prepared in 0.7% NaCl (25 μl per abdomen), then injected into recipient pupae between the second and third abdominal segments using pulled capillary needles attached to an oil-filled Hamilton syringe. The volume injected was standardised according to recipient pupal size: 0.5 μl was employed for *A. novemdecimpunctata *and *P. quatuordecimpunctata*, 1 μl for *A. bipunctata*, *A. decempunctata *and *E. quadripustulatus*, 1.25 μl for *C. quatuordecimguttata *and 1.5 μl for *C. septempunctata *and *H. quadripunctata*. Homogenate infectivity was confirmed on each occasion by injection back into uninfected *A. bipunctata *pupae. Whole body homogenate of *Acyrthosiphon pisum *adults carrying their *Spiroplasma *symbiont were similarly prepared and injected into *A. bipunctata *pupae. Aphids were derived from a laboratory clone collected in Bayreuth, Germany in 2001.

After pupae eclosed, females were maintained at 21°C on a mixed diet of aphids (*Acyrthosiphon pisum*) and artificial food [47] for one month before breeding to allow bacterial replication; males were discarded. Most species were bred directly following this incubation period. However, *C. quatuordecimguttata*, *E. quadripustulatus *and most *C. septempunctata *require diapause to initiate oviposition [51] therefore females were stored in an incubator at 4–8°C (24 hr dark) for around three months before breeding. Injection caused over 50% pupal mortality, further deaths occurred between eclosion and breeding and many females failed to oviposit: several hundred pupae of each species were injected to derive breeding samples. Surviving females were fed aphids, mated and allowed to oviposit at 21°C. Egg hatch rates (proportion hatched) and progenic sex ratios (proportion male) were assessed. The significance of offspring sex ratio deviations from 1:1 was calculated using one-tailed Fisher exact tests. PCR assays were used to determine if *Spiroplasma *bacteria were present in the injected females, their eggs and their offspring.

### Molecular techniques

Molecular methods followed Tinsley and Majerus [20]. In short, DNA was extracted by incubating samples in buffer containing Chelex-100 resin, DTT and Proteinase K, then used in diagnostic PCR reactions employing primers specific for the following bacterial taxa: *Spiroplasma *(MGSO – Ha-In-1 [33,52]), *Wolbachia *(wps81f – wsp691r [53]), *Rickettsia *(RSSUF – RSSUR [50]) and Flavobacteria (FL1 – FL2 [54]). Products were identified by agarose gel electrophoresis. Presence of amplifiable template was verified using mitochondrial DNA primers C1-J-2630 and TL2-N-3014 [55] and universal invertebrate ribosomal primers BD1 and 4S [56,57]; if product was lacking the sample was discarded.

For phylogenetic analysis we investigated two conserved sections from the 3' ends of the 16S and 12S mitochondrial ribosomal subunits of all host species. These were amplified using primer pairs N1-J-12585 – LR-N-13398 (16S) and SR-J-14233 – SR-N-14588 (12S) [58]. Products were purified using Sigma GeneElute™ columns and sequenced directly using the PCR primers by MWG-Biotech (EMBL accession numbers [AM779598] – [AM779613]). We discarded the data from the 3' region of the 16S fragment which spanned the 5' end of the ND1 gene and the leucine tRNA and combined the remaining sequence from the two rDNA genes. A sequence alignment was constructed using CLUSTALW and manually edited, then a tree was constructed using the neighbour joining method in MEGA version 4.0 [59] and evaluated by maximum composite likelihood, employing pair-wise gap deletion and a gamma distributed rate variation parameter of 0.255 (calculated in PAUP* version 4.0b8 [60]). Robustness of this tree was tested by performing 1000 bootstrap replicates. Genetic distances of novel hosts from *A. bipunctata *were calculated in MEGA using the parameter options above. Correlations of the species means for bacterial transmission rate and offspring sex ratio with genetic distance were determined non-parametrically using Spearman rank tests due to non-normality of dependent variables.

## Authors' contributions

MCT collected insect samples from the field, performed injections and conducted molecular work. MCT and MENM collaboratively designed the study, reared ladybirds in the laboratory and interpreted the results. MCT drafted the paper. Both authors read and approved the final manuscript.
